# Effects of syllable boundaries in Tibetan reading

**DOI:** 10.1038/s41598-022-25759-1

**Published:** 2023-01-06

**Authors:** Danhui Wang, Man Zeng, Han Zhao, Lei Gao, Shan Li, Zibei Niu, Xuejun Bai, Xiaolei Gao

**Affiliations:** 1 Plateau Brain Science Research Center, Tibet University, Lhasa, 850000 China; 2 Faculty of Psychology, Tianjin Normal University, Tianjin, 300387 China

**Keywords:** Neuroscience, Psychology

## Abstract

Interword spaces exist in the texts of many languages that use alphabetic writing systems. In most cases, interword spaces, as a kind of word boundary information, play an important role in the reading process of readers. Tibetan also uses alphabetic writing, its text has no spaces between words as word boundary markers. Instead, there are intersyllable tshegs (“ 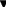
”), which are superscript dots. Interword spaces play an important role in reading as word boundary information. Therefore, it is interesting to investigate the role of tshegs and what effect replacing tshegs with spaces will have on Tibetan reading. To answer these questions, Experiment 1 was conducted in which 72 Tibetan undergraduates read three-syllable-boundary conditions (normal, spaced, and untsheged). However, in Experiment 1, because we performed the experimental operations of deleting tshegs and replacing tshegs, the spatial information distribution of Tibetan sentences under different operating conditions was different, which may have a certain potential impact on the experimental results. To rule out the underlying confounding factor, in Experiment 2, 58 undergraduates read sentences for both untsheged and alternating-color conditions. Overall, the global and local analyses revealed that tshegs, spaces, and alternating-color markers as syllable boundaries can help readers segment syllables in Tibetan reading. In Tibetan reading, both spaces and tshegs are effective visual syllable segmentation cues, and spaces are more effective visual syllable segmentation cues than tshegs.

## Introduction

Interword spaces exist in the texts of many languages that use alphabetic writing systems. In most cases (except for some English compound words that have spaces between their constituents, such as ice cream), interword spaces, as a kind of word boundary information, play an important role in the reading process of readers. Taking English and Spanish reading as an example, interword spaces ensure the reader’s lexical recognition and reading speed. If interword spaces are artificially deleted or replaced in other forms, the reader’s lexical recognition time will increase and the reading speed will decrease^1–9^. This shows that in languages with interword spaces, such as English and Spanish, interword spaces help readers to segment words, thus ensuring the normal reading of readers. In some languages, such as Thai and Chinese, texts do not contain interword spaces. Researchers have examined the role of spaces in reading text by adding interword spaces^10–15^. For Thai, studies have found that adding interword spaces in texts can improve the reading speed of native Thai speakers^10^. Some studies have also found that adding spaces between words in Thai texts can not improve the reading speed of native Thai speakers, but it can promote Thai lexical recognition^11^. For Chinese, studies have found that adding interword spaces in Chinese texts can promote Chinese reading^12^. In contrast, some studies have also found that adding interword spaces in Chinese texts does not promote Chinese reading, but can promote Chinese lexical recognition^13–15^. To summarize, we speculate that interword space—whether it exists naturally in the text or has been added to the text artificially—can help readers segment words, thereby helping readers read.

Tibetan is a language that uses an alphabetic writing system, consisting of 30 consonants and four vowels. In Tibetan text, a syllable is the basic writing unit, and the Tibetan lexical comprises one or more syllables^16,17^. For example, the Tibetan syllable “
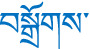
” consists of six consonants and one vowel. Among them, “
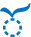
” is the superscribed vowel, “
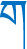
” is the base consonant letter, “
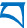
” is the prefix consonant letter, “
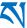
” is the superscribed consonant letter, “
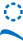
” is the subjoined consonant letter, “
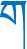
” is the suffix consonant letter, and “
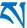
” is the post suffix consonant letter. A Tsheg, “
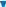
”, acts as a separator between syllables^18^. There are similarities between Tibetan and English in terms of language type and orthographic transparency (orthographic transparency refers to the degree of correspondence between a grapheme and phoneme, that is, the degree of knowing the pronunciation by the spelling)^19^. First, for language types, both Tibetan and English use alphabetic writing systems. Second, for orthographic transparency, both English and Tibetan are languages with relatively opaque orthography^19–23^. Tibetan differs from English in terms of script structure and boundary information. First, for script structure, English words are made up of letters horizontally. But Tibetan words can be composed of horizontally and vertically arranged letters^18^. Second, for boundary information, unlike English, there are no interword spaces as word boundary information in Tibetan. Instead, tshegs (“'.”) are used as syllable-boundary markers^16–18^. Although spaces and tshegs are different in form, the former is usually used as the word segmentation marker, and the latter is the syllable segmentation marker, both of which provide boundary information to segment text. Comparisons of Tibetan and English characteristics are shown in Table 1.

**Table 1:** Comparisons between Tibetan and English.

Language	Linguistic type		Script structure		Boundary information		Orthographic transparency	
	Alphabetic writing system	Logographic writing system	Horizontal array	Vertical array	Tsheg	Space	Transparent	Opaque
Tibetan	Yes	No	Yes	Yes	Yes	No	No	Yes
English	Yes	No	Yes	No	No	Yes	No	Yes

**Figure 1:**
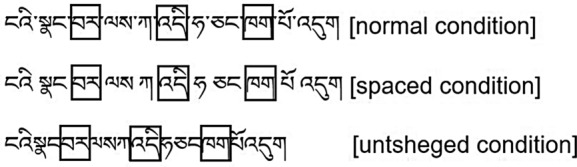
A sample Tibetan sentence displayed with different visual conditions (the boxes mean areas of interest). Translation: Today is the first day of the new semester.

In English and Spanish, interword spaces can be used as word boundaries to help readers segment words, thereby ensuring the normal reading of readers^1–9^. So can tshegs—which are also boundary information—as syllable boundaries help readers to segment syllables, thus ensuring the normal reading of readers? Moreover, if tshegs are replaced with spaces, what effect will it have on Tibetan reading? When discussing the role of spaces in English and Spanish reading, researchers usually delete or replace spaces, so we can also try to use this line of thinking when looking for answers to the above questions. First, by comparing the normal text with untsheged (removed tshegs) condition, we investigate whether tshegs can help readers segment syllables in Tibetan reading, just as spaces can help readers segment words in English and Spanish reading. Second, by contrasting the spaced condition with untsheged condition, we investigate whether replacing tshegs with spaces can also help readers segment syllables while reading. Third, by comparing the normal text with spaced condition, we investigate whether spaces are more effective visual syllable segmentation cues than tshegs. Based on this, we conducted Experiment 1, in which we adopted eye-tracking technology. We used the eye-tracking technology for the following two reasons: first, the eye-tracking technology has strong anti-interference and high ecological validity, and can record the reader's eye movement data in real time; second, in eye movement data analysis, we can not only analyze the eye movement data of the entire sentence but also divide visual objects into different areas of interest (AOI) according to the research needs. From this, the eye movement data in each area of interest can be obtained^24,25^. In the eye movement study of reading, researchers often select target areas for data analysis based on the needs of the experiment. These target areas are called areas of interest. An area of interest can be a character, a syllable, a word, or a phrase. The visual size and spatial information distribution of the area of interest remained consistent across different experimental manipulation conditions in the same sentence with the same content. The visual size and spatial information distribution of the area of interest “
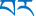
”, for instance, in the sentence “
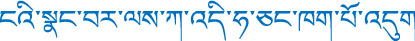
” (Today is the first day of the new semester), were constant across the three experimental manipulation conditions (normal condition, spaced condition and untsheged condition). A sample is provided in Fig. 1. Additionally, the area of interest does not appear at the beginning and end of the sentences (avoiding the interference of fixation points at the beginning and end of reading)^25,26^.

## Experiment 1

In this experiment, we used eye-tracking technology to investigate three questions by manipulating three presentation conditions (normal, spaced, and untsheged). First, we examined whether tshegs can help readers to segment syllables during Tibetan reading, thus ensuring the normal reading of readers. Second, whether replacing tshegs with spaces can also help readers segment syllables during Tibetan reading. Third, whether spaces are more effective visual syllable segmentation cues than tshegs during Tibetan reading.

### Participants

A total of 72 Tibetan university undergraduates participated in the eye-tracking experiment (30 males, 42 females; *M*_age_ = 20.68 years, *SD* = 1.54). They were native Tibetan speakers with normal (or corrected-to-normal) vision, and were right-handed. None of them had dyslexia or deficits related to intelligence. Additionally, they had no color vision deficiency or physical or mental diseases. All participants voluntarily participated in the experiment and received rewards after the experiment. Informed consent was obtained from all participants before the experiment.

## Materials and method

### Selecting and preparing the experimental materials

Referring to a prior study^16^, 136 sentences with 18–23 horizontal letter-spaces were selected and edited appropriately from senior primary Tibetan textbooks and extracurricular reading materials of a comparable level. None of these sentences had semantic or syntactic ambiguity, and all sentences were declarative. It is worth noting that, referring to prior studies, when a Tibetan letter has a viewing angle of about 0.6°, the perceptual span of Tibetan reading is up to three horizontal letter-spaces to the left of the fixation point, and up to 8 horizontal letter-spaces to the right of the fixation point, spanning across up to 12 horizontal letter-spaces^16,18^. In this experiment, a Tibetan letter also has a viewing angle of about 0.6°, and the sentence length is 18–23 horizontal letter-spaces, much larger than 12 horizontal letter-spaces. Therefore, the sentence length used in this experiment is longer than the perceptual span of Tibetan reading, and readers must read from left to right.

### Evaluating experimental materials

After compiling the sentences, we asked 15 Tibetan undergraduates to rate their difficulty on a 5-point scale (1 = very easy, 5 = very difficult, *M* = 1.21, *MED* = 1.2, *SD* = 0.12). Moreover, 15 Tibetan undergraduates were asked to rate naturalness on a 5-point scale of naturalness: (1 = entirely unnatural, 5 = entirely natural, *M* = 4.55, *MED* = 4.53, *SD* = 0.20). These undergraduates did not participate in the formal experiment. Finally, 72 sentences were selected as formal experimental materials.

### Design

**Figure 2:**
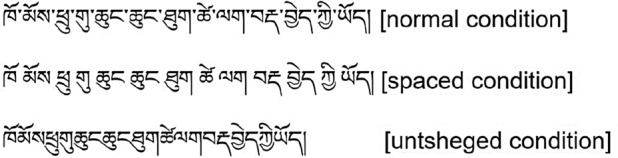
A sample Tibetan sentence displayed with different visual conditions. Translation: She greets children when she sees them.

We used a single-factor within-subject design with three levels (visual cues: normal condition, inter-syllable spaced condition, and untsheged condition). The independent variable was the visual cue and the dependent variables were the eye movement measures. The experiment was divided into three blocks, and each block contained 72 formal experimental sentences, including 24 sentences each in the normal, inter-syllable spaced, and untsheged conditions. The experimental conditions were rotated across blocks according to Latin Square, and sentences in each block were presented randomly. Each participant was asked to read one block. Before the formal experiment, the participants practiced nine sentences (three practice sentences in each condition). In addition, 24 yes/no comprehension questions (half of “yes”) were set according to the content of the formal experimental sentences to ensure that participants read carefully and correctly. A total of 81 sentences were read by each participant. Examples of experimental materials are presented in Fig. 2.

### Apparatus

Eye movements were recorded using an SR Research EyeLink 1000 Plus eye tracker (sampling rate = 1000 Hz). EyeLink default settings for cognitive research were used in the data acquisition (saccade velocity threshold: 30°/sec; saccade acceleration threshold: 8000°/sec^2^; saccade motion threshold: 0.1°)^27,28^. All stimuli were presented at a distance of 65 cm from the participants on a 21-inch CRT monitor (SONY MuLtiscanG520) with a resolution of 1024 × 768 pixels and refresh rate of 140 Hz. Each sentence was displayed in Microsoft Himalaya 32 fonts on a white background. Each Tibetan letter was horizontally occupied by 15 pixels and subtended approximately 0.6° of visual angle.

### Procedure

Each participant was individually tested. After entering the laboratory, the participants were asked to familiarize themselves with the environment, sit down as required, and read the instructions. Subsequently, the experimenter explained the instructions again to ensure that the participants correctly understood the experimental procedure and emphasized that they should keep their heads as still as possible during the experiment. Prior to the experiment, the viewing positions were calibrated using a 3-point grid (error < 0.25°)^16^. After successful calibration, the experiment was initiated. Calibration was checked after each trial, and participants were recalibrated whenever necessary. To avoid fatigue, the experiment did not exceed 20 min. If the participant felt tired during the experiment, they could choose to end the experiment or continue after a short rest. If the participant continued the experiment after a rest, we needed to recalibrate.

### Results

Of the 72 participants, three were excluded from all subsequent analyses because of poor performance (< 70%) on sentence reading comprehension questions. For the remaining participants, the average comprehension accuracy was 93.06%, indicating that the participants read carefully and fully understood the sentences. Referring to existing studies^13,17,29,30^, the data were analyzed according to the following four criteria. First, data in which subjects pressed a key too early or pressed a key incorrectly resulting in interrupted sentence presentation were excluded. Second, invalid data due to tracking loss were excluded; third, data with fixation durations shorter than 80 ms or longer than 1200 ms were excluded. Finally, all the eye movement measures above or below three standard deviations from the mean were also excluded. In total, 12.74% of the data were removed from the analysis.

Estimates were based on linear mixed-effects models (LMMs) using the lmer program of the lme4 package (Version 1.1–26)^31^ in the R environment for statistical computing (Version 4.1.1)^32^. For the subsequent analysis, these measures were log-transformed to achieve a more normal distribution. Visual cues were specified as a fixed factor. We included crossed random intercepts for participants and items as well as random slopes for participants in our models unless they failed to converge^33^.

### Global analysis

In the global analysis, based on the whole sentence under different experimental conditions, we analyzed different eye movement measures^14,26^, and we chose the following seven measures: mean fixation duration (average fixation duration in all fixated points in a sentence), mean saccadic length (the average length of all the saccades made on a sentence), number of progressive saccades (saccades made in a left-to-right direction), total number of fixations (total number of fixations made on a sentence), number of regressive saccades (saccades made from right to left), reading speed (reading syllables in a minute, dividing the number of syllables by the total reading time), and total sentence reading time (the sum of all the fixations and saccades made during sentences reading). Table 2 presents the descriptive statistical results of the eye movement measures for different visual cues.

**Table 2:** Eye movement measures for the global analysis across conditions (standard deviations in parentheses).

Measure	Normal	Spaced	Untsheged
Mean fixation duration (ms)	247 (28)	245 (29)	267 (29)
Mean saccadic length	2.10 (0.33)	2.11 (0.34)	1.68 (0.28)
Number of progressive saccades	9.98 (2.58)	9.68 (2.30)	9.89 (2.52)
Total number of fixations	15.01 (4.81)	14.72 (4.61)	15.61 (5.29)
Number of regressive saccades	3.69 (1.39)	3.62 (1.41)	3.74 (1.56)
Reading speed (syllable/min)	262 (74)	272 (79)	248 (78)
Total sentence reading time (ms)	5866 (1865)	5732 (2102)	6379 (2351)

As shown in Table 2, mean fixation duration was shorter in both the normal condition (*b* = −0.076, *SE* = 0.004, *t* = −19.441, *p* < 0.001, *d* = 0.537) and spaced condition (*b* = −0.088, *SE* = 0.004, *t* = −22.408, *p* < 0.001, *d* = 0.622) than in the untsheged condition. Mean fixation duration was significantly longer in the normal condition than in the spaced condition (*b* = 0.012, *SE* = 0.004, *t* = 3.013, *p* = 0.003, *d* = 0.186). Mean saccadic length was significantly longer in the normal (*b* = 0.225, *SE* = 0.007, *t* = 32.261, *p* < 0.001, *d* = 0.865) and spaced conditions (*b* = 0.231, *SE* = 0.007, *t* = 32.970, *p* < 0.001, *d* = 0.888) than in the untsheged condition. However, no significant difference was observed between the normal and spaced conditions (*b* = 0.006, *SE* = 0.007, *t* = 0.822, *p* = 0.411, *d* = 0.023). Number of progressive saccades showed no significant difference between the untsheged and normal conditions (*b* = 0.010, *SE* = 0.008,* t* = 1.328, *p* = 0.184, *d* = 0.031), as well as between the untsheged and spaced conditions (*b* = −0.015, *SE* = 0.008, *t* = −1.923, *p* = 0.053, *d* = 0.046), while number of progressive saccades was significantly higher in the normal condition than in the spaced condition (*b* = 0.025, *SE* = 0.008, *t* = 3.272, *p* = 0.001, *d* = 0.216). Number of regressive saccades was similar between the normal condition and untsheged condition (*b* = −0.009, *SE* = 0.019, *t* = −0.469, *p* = 0.639, *d* = 0.014). Number of regressive saccades was similar between the spaced condition and untsheged condition (*b* = 0.021, *SE* = 0.019, *t* = 1.106, *p* = 0.269, *d* = 0.132). Number of regressive saccades was similar between the normal condition and spaced condition (*b* = −0.030, SE = 0.019, *t* = −1.583, *p* = 0.114, *d* = 0.046). Total number of fixations was significantly lower in the normal condition (*b* = -0.031, *SE* = 0.010, *t* = −3.213, *p* = 0.001, *d* = 0.207) and spaced condition (*b* = −0.051, *SE* = 0.010, *t* = −5.313, *p* < 0.001, *d* = 0.226) than in the untsheged condition. Total number of fixations was significantly higher in the normal condition than in the spaced condition (*b* = 0.020, *SE* = 0.010, *t* = 2.121, *p* = 0.034, *d* = 0.150). Reading speed was significantly faster in the normal (*b* = 0.077, *SE* = 0.009, *t* = 8.228, *p* < 0.001, *d* = 0.248) and spaced conditions (*b* = 0.103, *SE* = 0.009, *t* = 10.985,* p* < 0.001, *d* = 0.298) than in the untsheged condition. Reading speed was significantly slower in the normal condition than in the spaced condition (*b* = −0.026, *SE* = 0.009, *t* = −2.786, *p* = 0.005, *d* = 0.150). Total sentence reading time was significantly shorter in the normal (*b* = −0.074, *SE* = 0.010, *t* = −7.600, *p* < 0.001, *d* = 0.243) and spaced condition (*b* = −0.105, *SE* = 0.010, *t* = −10.646, *p* < 0.001, *d* = 0.302) than in the untsheged condition. Total sentence reading time was significantly longer in the normal condition than in the spaced condition (*b* = 0.030, *SE* = 0.010, *t* = 3.084, *p* = 0.002, *d* = 0.201).

### Local analysis

**Figure 3:**
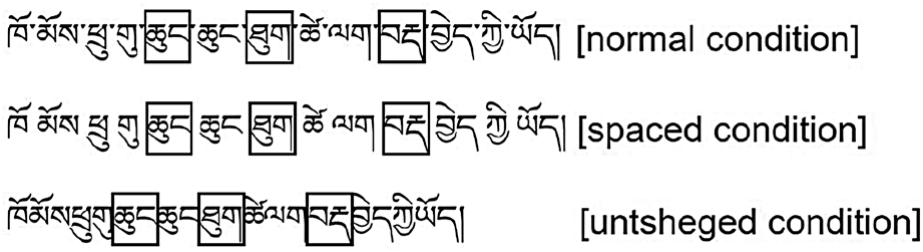
An illustration of local analysis based on areas of interest in different conditions (the boxes mean areas of interest). Translation: She greets children when she sees them.

In addition to the global analysis, we also conducted a local analysis of the data within our selected areas of interest. In this experiment, three syllables with two horizontal letter-spaces were randomly selected as areas of interest in each experimental sentence, as shown in Fig. 3, which presents the areas of interest delineated by the local analysis. Under different conditions, the visual size and spatial information distribution of each area of interest are consistent, and the area of interest does not appear at the beginning and end of the sentences^25,26^. Referring to previous studies, the measures of local analysis include: first fixation duration (the duration of the first fixation on a syllable), gaze duration (the sum of all first-pass fixations on the target region before moving to another region), total fixation time (the sum of all fixations on a syllable, including regressions), number of first-pass fixations (the sum of all first-pass fixations within a specified region, plus any within-zone regressions up to and including the final fixation), and total number of fixations (total number of times that a target syllable was fixated during any given trial)^26,34^. Table 3 shows the descriptive statistical results of the eye movement measures for different visual cues.

**Table 3:** Means (and standard deviations) of eye movement measures for local analysis.

Measures	Normal	Spaced	Untsheged
First fixation duration (ms)	252 (33)	250 (33)	269 (35)
Gaze duration (ms)	281 (45)	280 (43)	316 (52)
Total fixation time (ms)	409 (118)	399 (111)	479 (149)
Number of first pass fixations	1.11 (0.09)	1.11 (0.08)	1.17 (0.12)
Total number of fixations	1.62 (0.36)	1.60 (0.36)	1.79 (0.43)

The results showed that: first fixation duration was significantly shorter in both the normal (*b* = −0.064, *SE* = 0.008, *t* = −7.853, *p* < 0.001, *d* = 0.268) and spaced conditions (*b* = −0.071, *SE* = 0.008, *t* = −8.665, *p* < 0.001, *d* = 0.287) than in the untsheged condition, while normal and spaced conditions were read similarly (*b* = 0.007, *SE* = 0.008, *t* = 0.850, *p* = 0.396, *d* = 0.018). Gaze duration was significantly shorter in both the normal (*b* = −0.106, *SE* = 0.010, *t* = −11.059, *p* < 0.001, *d* = 0.338) and spaced conditions (*b* = −0.110, *SE* = 0.010, *t* = −11.492, *p* < 0.001, *d* = 0.348) than in the untsheged condition. By contrast, there was no significant difference between the normal and spaced conditions (*b* = 0.005, *SE* = 0.010, *t* = 0.479, *p* = 0.632, *d* = 0.011). Total fixation time was significantly shorter in both the normal (*b* = −0.147, *SE* = 0.012, *t* = −12.056, *p* < 0.001, *d* = 0.349) and spaced conditions (*b* = −0.168, *SE* = 0.012, *t* = −13.743, *p* < 0.001, *d* = 0.385) than in the untsheged condition. However, no significant difference was observed between the normal and spaced conditions (*b* = 0.021, *SE* = 0.012, *t* = 1.744, *p* = 0.081, *d* = 0.036). Number of first-pass fixations was significantly lower in both the normal (*b* = −0.039, *SE* = 0.005, *t* = −7.271, *p* < 0.001, *d* = 0.262) and spaced conditions (*b* = −0.042, *SE* = 0.005, *t* = −7.933, *p* < 0.001, *d* = 0.274) than in the untsheged condition, while there was no significant difference between the normal and spaced conditions (*b* = 0.004, *SE* = 0.005, *t* = 0.692, *p* = 0.489, *d* = 0.017). Total number of fixations in both normal (*b* = −0.084, *SE* = 0.010, *t* = −8.186, *p* < 0.001, *d* = 0.273) and spaced conditions (*b* = −0.093, *SE* = 0.010, *t* = −9.071, *p* < 0.001, *d* = 0.291) were significantly lower than those in the untsheged condition. However, there was no significant difference between the normal and spaced conditions (*b* = 0.009, *SE* = 0.010, *t* = 0.915, *p* = 0.360, *d* = 0.019).

### Interim discussion

Global analysis showed that, compared with the untsheged condition, the normal condition produced shorter mean fixation duration and total sentence reading time, larger mean saccadic length, lower total number of fixations, and faster reading speed. Local analysis indicated that, compared with the untsheged condition, the normal condition resulted in shorter first fixation duration, gaze duration, and total reading time, in addition to a lower number of first-pass fixations and total number of fixations. The results showed that tshegs, as syllable boundaries, can help readers segment syllables during reading, thus ensuring the normal reading of readers. they are effective visual syllable segmentation cues in Tibetan syllable recognition and sentence reading. As a result, the first question was answered. Next, in the global measures, compared to the untsheged condition, the spaced condition resulted in shorter mean fixation time and total sentence reading time, lower total number of fixations, and faster reading speed. From the local analysis, we can see that the spaced condition produced shorter first fixation duration, gaze duration, and total fixation time, as well as a lower number of first-pass fixations and total number of fixations, compared to the untsheged condition. The above results reveal that spaces, as syllable boundaries, can help readers segment syllables during reading. As a result, the second question was answered. From the results of the global analysis, the spaced condition produced shorter mean fixation time and total sentence reading time, lower number of progressive saccades and total number of fixations, and faster reading speed relative to normal conditions. The results showed that spaces are more effective visual syllable segmentation cues than tshegs during Tibetan reading. Thus, the third question was answered. However, the local analysis found that the difference between the normal and spaced conditions was not significant in all indicators, demonstrating that the tshegs and spaces play the same role in Tibetan syllable recognition. In other words, spaces did not promote syllable recognition in Tibetan reading compared to tshegs. To summarize, we found that replacing tshegs in the Tibetan text with spaces did not improve the syllable recognition in readers’ Tibetan reading, but improved their reading of Tibetan sentences. For this result, we speculate the reason may be that Tibetan readers may not process syllables as a whole during Tibetan reading; in other words, syllables may not be the basic information processing units in Tibetan reading. This experimental manipulation of replacing a tsheg with a space may facilitate the identification of the true basic information processing unit in Tibetan reading, which further facilitates Tibetan sentence reading.

Similar to previous studies, removing the intrinsic space in the text elicited a lower reading efficiency^7,8^. Likewise, the current study removed the intrinsic tshegs in written Tibetan and found that readers also had difficulty processing the text, which illustrated that the intrinsic tshegs serving as syllable boundary information could ensure readers to read Tibetan text. The results of Experiment 1 are also similar to the results of other studies, that under the condition of replacing spaces with other forms, the reading efficiency of readers decreases significantly^4,6,9^. This study replaced tshegs in Tibetan text with spaces in the opposite way, and the results showed that spaces can improve readers’ Tibetan reading efficiency, suggesting that spaces are better visual syllable segmentation cues than tshegs.

However, when tshegs were deleted or replaced with spaces in Tibetan, it introduced a limitation, which may have interfered with the experimental results to some extent. This is because the spatial information distribution of the text is modified for different presentation conditions. Specifically, compared to the normal and spaced conditions, the untsheged condition produced a shorter sentence length and higher information density per unit. Compared to the normal condition, the spaced condition produced a longer sentence length and smaller information density per unit. To further rule out this effect, we aimed to look for syllable boundaries that would provide clear boundary information for readers’ reading without affecting the spatial distribution of sentences. In previous studies, artificially removing or adding spaces to text, whether in languages with or without spaces, changed the spatial distribution of the sentences. Accordingly, the current researchers came up with the idea of alternating-color markers as visual cues that provide readers with boundary information without changing the spatial distribution of sentences.

The same color can integrate several parts into a whole, and different colors can segregate a whole into several parts^35^. Color alternation mark words are helpful in detecting, recognizing, and classifying, and can attract the readers’ perceptual attention^36,37^. Studies have found that in Italian reading, the reading efficiency is higher under the alternating-color condition (i.e., each touching word was marked with a different color) than the single-color condition^38–40^. In Spanish reading, the removal of interword spaces can be compensated by color alternation markers for saccade target selection^41^. In Chinese reading, word boundary information with alternating colors can help native Chinese speakers as well as Chinese second language speakers to read Chinese texts^42–45^ . From these findings, we have learned that in many languages, alternating-color marks as word boundaries can help readers segment words, which in turn helps them read.

Next, we conducted Experiment 2. To ensure the same distribution of sentence spatial information, we set two presentation conditions: alternating-color condition (each adjacent syllable is marked with a different color) and untsheged condition. By comparing the alternating-color condition and the untsheged condition, we investigate whether alternating-color markers can help Tibetan readers segment syllables in Tibetan reading.

## Experiment 2

Experiment 2 also employed eye-tracking technology. Two conditions were set to investigate whether the alternating-color markers as syllable boundaries can help Tibetan readers segment syllables in Tibetan reading.

### Participants

Fifty-eight Tibetan university undergraduates participated in the study (27 males, 31 females; *M*_*age*_ = 19.93 years, *SD* = 1.06). These participants did not participate in Experiment 1. The selection criteria of the participants were the same as in Experiment 1. All participants voluntarily participated in the experiment and received rewards after the experiment. Informed consent was obtained from all participants before the experiment.

### Materials and method

**Figure 4:**
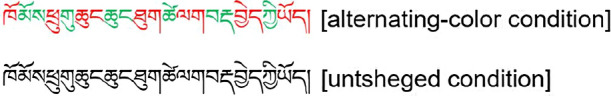
A sample of experimental materials. Translation: She greets with the children when she sees them.

The materials were the same as in Experiment 1, with the only difference being the manipulation of spatial information. The experimental design was a single-factor within-subjects design with two conditions (alternating-color, untsheged). The experiment was divided into two blocks, each containing 72 formal experimental sentences, including 36 sentences for each condition. The experimental conditions were rotated across blocks according to Latin Square, and sentences in each block were presented randomly. Each participant read one block. Before the formal experiment, six sentences were set up for the participants to practice, and there were three practice sentences in each condition. Additionally, to ensure that the participants read carefully and correctly, 24 yes/no comprehension questions (half of “yes”) according to the content of the formal experimental sentences were set. Each participant was required to read 78 sentences. An example of the experimental setup is shown in Fig. 4.

### Apparatus, procedure and measures

Identical to Experiment 1.

### Results

Two participants with a comprehension accuracy of less than 70% were excluded. The average comprehension accuracy was 91.96%, indicating that participants read carefully and understood the sentences. The data exclusion standards were in line with Experiment 1, and the rejected invalid data accounted for 12.44% of the total data. The eye movement measures and data analysis methods were the same as those in Experiment 1.

### Global analysis

The descriptive statistical results of eye movement measures based on global analysis for different visual cues are shown in Table 4.

**Table 4:** Means (and standard deviations) of eye movement measures based on global analysis.

Measure	Alternating-color	Untsheged
Mean fixation duration (ms)	255 (29)	261 (29)
Mean saccadic length	1.79 (0.24)	1.70 (0.25)
Number of progressive saccades	8.96 (1.83)	9.40 (1.96)
Total number of fixations	13.71 (3.32)	14.65 (3.57)
Number of regressive saccades	3.36 (1.02)	3.57 (1.07)
Reading speed (syllable/min)	278 (74)	257 (65)
Total sentence reading time (ms)	5583 (1641)	5939 (1561)

We found that mean fixation duration was significantly shorter in the alternating-color condition than in the untsheged condition (*b* = −0.024, *SE* = 0.005, *t* = −4.986, *p* < 0.001, *d* = 0.250). Mean saccadic length was significantly longer in the alternating-color condition than in the untsheged condition (*b* = 0.049, *SE* = 0.007, *t* = 7.380, *p* < 0.001, *d* = 0.300). Number of progressive saccades in the alternating-color condition was significantly lower than in the untsheged condition (*b* = −0.048, *SE* = 0.008, *t* = −5.769, *p* < 0.001, *d* = 0.253). Total number of fixations in the alternating-color condition was significantly lower than in the untsheged condition (*b* = −0.062, *SE* = 0.009, *t* = −6.911, *p* < 0.001, *d* = 0.262). Number of regressive saccades in the alternating-color condition was significantly lower than in the untsheged condition (*b* = −0.050, *SE* = 0.018, *t* = −2.748, *p* = 0.006, *d* = 0.179). Reading speed was significantly faster in the alternating-color condition than in the untsheged condition (*b* = 0.077, *SE* = 0.012, *t* = 6.584, *p* < 0.001,* d* = 0.248). Total sentence reading time was significantly shorter in the alternating-color condition than in the untsheged condition (*b* = −0.067, *SE* = 0.011, *t* = −5.866, *p* < 0.001, *d* = 0.234).

### Local analysis

**Figure 5:**
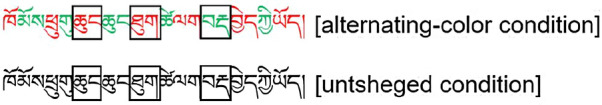
An illustration of local analysis based on areas of interest in different conditions (the boxes mean areas of interest). Translation: She greets with the children when she sees them.

In Experiment 2, in addition to the global analysis, we also conducted a local analysis of the data within our selected areas of interest. The criteria for selecting areas of interest were the same as in Experiment 1. Figure 5 shows the areas of interest for the local analysis. Table 5 shows descriptive statistical results of eye movement measures for different visual cues.

**Table 5:** Means (and standard deviations) of eye movement measures based on global analysis.

Measures	Alternating-color	Untsheged
First fixation duration(ms)	256 (34)	262 (34)
Gaze duration (ms)	293 (48)	301 (44)
Total fixation time (ms)	420 (107)	453 (111)
Number of first pass fixations	1.13 (0.08)	1.14 (0.09)
Total number of fixations	1.63 (0.32)	1.73 (0.36)

Local analysis showed that first fixation duration was significantly shorter in the alternating-color condition than in the untsheged condition (*b* = −0.018, *SE* = 0.008, *t* = −2.363, *p* = 0.018, *d* = 0.146). Gaze duration was significantly shorter in the alternating-color condition than the untsheged condition (*b* = −0.025, *SE* = 0.009, *t* = −2.811, *p* = 0.005, *d* = 0.150).Total fixation time was significantly shorter in the alternating-color condition than in the untsheged condition (*b* = −0.070, *SE* = 0.012, *t* = −5.624, *p* < 0.001, *d* = 0.209). Number of first-pass fixations was significantly lower in the alternating-color condition than in the untsheged condition (*b* = −0.009, *SE* = 0.005,* t* = −2.030, *p* = 0.142, *d* = 0.141). Total number of fixations was significantly lower in the alternating-color condition than in the untsheged condition (*b* = −0.053, *SE* = 0.009, *t* = −5.594, *p* < 0.001, *d* = 0.207).

### Interim discussion

According to the global analysis, compared with the untsheged condition, the alternating-color condition resulted in shorter mean fixation duration and total sentence reading time, as well as a lower number of progressive saccades, total number of fixations, and number of regressive saccades. There was also a larger mean saccadic length and faster reading speed in the alternating-color condition than in the untsheged condition. According to the local analysis, the first fixation duration, gaze duration, and total fixation time were shorter, and the number of first-pass fixations and total number of fixations were lower in the alternating-color condition than in the untsheged condition. The combined results of global and local analyses implied that alternating-color markers can help Tibetan readers segment syllables in Tibetan reading.

In studies of Italian and Spanish reading, researchers segmented the word boundaries in the text using different colors and found that text with alternating colors was easier to read than text with a single color. This shows that, in Italian as well as Spanish reading, alternating-color markers as word boundaries can help readers segment words^38–41^. This research effectively divided the syllable boundaries in Tibetan texts using different colors. The results showed that the total sentence reading time under the alternating-color condition is shorter than that under the untsheged condition. This also demonstrates that alternating-color markers as syllable boundaries can help Tibetan readers segment syllables in Tibetan reading.

### General discussion

Two experiments were conducted in this study to examine the role of syllable boundaries in Tibetan reading. Experiment 1 examined three questions. First, we examined whether tshegs can help readers to segment syllables during Tibetan reading, thus ensuring the normal reading of readers. Second, whether replacing tshegs with spaces can also help readers segment syllables during Tibetan reading. Third, whether spaces are more effective visual syllable segmentation cues than tshegs during Tibetan reading. We found that tshegs and spaces can indeed help readers segment syllables during Tibetan reading and are effective visual syllable segmentation cues. Interestingly, replacing tshegs with spaces can improve the readers’ reading efficiency.This shows that spaces are more effective visual syllable segmentation cues than tshegs. This result may be due to the visual crowding effect when reading normal Tibetan texts. The crowding effect is defined as the detrimental effect of nearby items on visual object recognition^46^^.^ For example, in English reading, when letters are close to each other, the unit spatial information density of the text increases and features from multiple letters are combined. Thus, “visual crowding” occurred^47^. In Tibetan reading, compared with the normal condition, the spacing between syllables in the spaced condition is larger, which leads to a smaller information density per unit space under the spaced condition, thereby reducing the readers’ visual crowding, and further promotes the readers' Tibetan reading efficiency.

To rule out the potential effect of differences in the spatial information distribution of sentences, we conducted Experiment 2, which examined whether alternating-color markers can help Tibetan readers to segment syllables. We found that alternating-color markers as syllable boundaries also played a role in helping readers segment syllables. However, to ensure that the spatial distributions of sentences under the two conditions are the same, Experiment 2 did not include setting the normal and spaced conditions, which makes us abandon the discussion of some interesting issues. For example, we cannot know whether the alternating-color markers have similar advantages as tshegs and spaces. That is, we cannot know whether alternating-color markers are also effective visual syllable segmentation cues. We can explore these questions in future research by comparing the normal condition with the alternating-color condition, and the spaced condition with the alternating-color condition.

In summary, this study found that tshegs, spaces, and alternating-color markers, which serve as syllable boundaries, can help readers segment syllables during Tibetan reading. Among them, spaces and tshegs are effective visual syllable segmentation cues in Tibetan reading. It is worth noting that replacing tshegs with spaces improves Tibetan reading efficiency, indicating that spaces are more effective visual syllable segmentation cues than tshegs in Tibetan reading. In this regard, in daily reading, we can improve the readers’ syllable segmentation ability by replacing the tshegs in Tibetan text with spaces, thereby improving their reading efficiency (Supplementary Information).

### Ethics approval

All procedures performed in studies involving human participants were in accordance with the ethical standards of the institutional and national research committee and with the 1964 Helsinki Declaration and its later amendments or comparable ethical standards. The study was approved by Ethics Committee of Psychology of Tibet Autonomous Region.

### Consent to participate

Informed consent was obtained from all individual participants included in the study.

## Supplementary Information

Supplementary Information.

## Data Availability

The datasets generated during and/or analyzed during the current study are available from the corresponding author on reasonable request.

## Code availability

The codes generated during and/or analyzed in the current study are available from the corresponding author on reasonable request.
